# Anti-cancer properties of *Escherichia coli* Nissle 1917 against HT-29 colon cancer cells through regulation of Bax/Bcl-xL and AKT/PTEN signaling pathways

**DOI:** 10.22038/ijbms.2020.43016.10115

**Published:** 2020-07

**Authors:** Siamak Alizadeh, Abolghasem Esmaeili, Yadollah Omidi

**Affiliations:** 1 Department of Cell and Molecular Biology & Microbiology, Faculty of Biological Science and Technology, University of Isfahan, Isfahan, Iran; 2 Research Center for Pharmaceutical Nanotechnology, Biomedicine Institute, Tabriz University of Medical Sciences, Tabriz, Iran; 3 Department of Pharmaceutics, Faculty of Pharmacy, Tabriz University of Medical Sciences, Tabriz, Iran

**Keywords:** AKT, Apoptosis, Cell signaling, Colon cancer, Escherichia coli Nissle1917

## Abstract

**Objective(s):**

Chemotherapies used to treat colon cancer might often fail due to the emergence of chemoresistance and side effects. *Escherichia coli *Nissle 1917 (EcN) is a beneficial probiotic, whose molecular mechanisms in the prevention of colon cancer are yet to be fully understood. The present study assessed the anti-cancer effects of EcN treatments in human colorectal cancer, HT-29 cell line, with the analysis of related mechanisms.

**Materials and Methods:**

The co-culture conditioned-media (CM) of EcN with adenocarcinoma HT-29 cells and heat-inactivated bacteria (HIB) were applied for the treatment of the HT-29 cells. To study the inhibition potential of CM and HIB on cancer cells, various cellular/molecular analyses were implemented, including DAPI-staining and DNA ladder assays, flow cytometry and Real-time quantitative PCR (qPCR), as well as Western blotting analyses.

**Results:**

Our results indicated that EcN could elicit apoptotic impacts on the colon cancer HT-29 cells by up-regulating *PTEN* and *Bax* and down-regulating *AKT1* and *Bcl-xL *genes.

**Conclusion:**

Based on our findings, EcN is proposed as a useful supplemental probiotic treatment against colon cancer.

## Introduction

Colon cancer is the third leading reason for cancer-associated death worldwide (1). Various determinant factors may be involved in the initiation and progression of colon cancer, including genetic and/or epigenetic mutations and environmental factors (e.g*.,* lifestyle and diet, health and disease, and pharmacotherapy regimen) (2). A wide range of studies supports the role of microbiota (i.e., colonic microflora) in the etiology of colorectal cancer, Therefore, the selective stimulation or deactivation of specific types of microbiota in the gut with beneficial roles in the body may influence the occurrence of colorectal cancer (3). These bacteria may intriguingly have a potential role in modulating, altering, and improving the gut microflora in colon cancer (3, 4). EcN is one of the most-studied probiotic strains, which may be used as edible probiotic for a broad range of gastrointestinal (GI) disorders, including ulcerative colitis (UC), inflammatory bowel disease (IBD), Crohn’s disease (CD), and other GIT-related diseases (4). Probiotics are nonpathogenic microorganisms that beneficially affect their hosts when used in an adequate amount (5). The unique features of the strain are (i) lack of virulence, (ii) antagonism toward the other pathogens, (iii) immunomodulatory properties, and (iv) survival factors and probiotic potential. These features make the strain a good and safe candidate for the treatment of GI disorders (4). Recently, EcN has successfully been used as one of the safest carriers for the therapeutic molecules both *in vitro *and* in vivo *(6). Although plenty of studies have been carried out in terms of the beneficial effects of EcN against intestinal diseases, still, limited information is available regarding the signaling pathways and molecular mechanisms involved in the anti-cancer effects of EcN in colorectal cancer (CRC). In this context, a deep understanding of the role of various signaling pathways that are always altered during cancer progression absolutely influences the successful treatment of cancers (7). Of the different signaling pathways, Bax and BCl-xL involved in the intrinsic apoptosis pathway have pivotal roles in cancer development and aggressiveness (8). Moreover, the PI3K/AKT signaling pathway and expression of *AKT* can particularly affect a variety of cellular processes, resulting in reduced apoptosis, enhanced cell growth, and proliferation. These signaling routes are negatively controlled by a tumor suppressor protein known as phosphatase and tensin homolog (PTEN) (9). Increased activation of *AKT* signaling and down-regulation of *PTEN* were reported to be associated with 60–70% of the human colon cancer patients (10). The human colon adenocarcinoma cell line HT29 represents a valuable model for attachment and mechanistic studies due to their similarities with enterocytes and mucin secretion ability. In fact, the mucosal layer created by this cell line seems to play a major role in the adhesion of bacteria or bacterial compounds to the epithelial surface (11). Therefore, this study was designed to investigate the potential of EcN or EcN-derived factors (CM and HIB) on the eradication of HT-29 colon cancer cells. Moreover, considering the importance of the aforementioned signaling paths and the therapeutic potential of EcN in GI disorders, we followed the controlling impact(s) of EcN on the initiation and progression of colorectal cancer throughout the investigation of *AKT*, *PTEN*, *Bax*, and *Bcl-xL *expressions. 

## Materials and Methods

***Reagents***

Colon adenocarcinoma cells (HT-29) were obtained from the National Cell Bank of Iran (Pasteur Institute, Tehran, Iran). RPMI-1640 media, fetal bovine serum (FBS) were purchased from Invitrogen^TM^ Gibco (Paisley, UK). TRIzol® Reagent, MMLV Reverse Transcriptase, RNase inhibitor, and dNTP were obtained from Thermo Fisher Scientific (Waltham, MA, USA). SYBR Green PCR Master Mix was obtained from Applied Biosystems (Foster City, CA, USA). The apoptosis detection kit, annexin V-fluorescein isothiocyanate (FITC), was purchased from eBioscience (Waltham, MA, USA). DAPI (4, 6-diamidino- 2-phenylindole) was attained from Sigma-Aldrich Co. (Poole, UK). PTEN (A2B1): sc-7974, AKT1 (B-1): sc-5298, Bax (B-9): sc-7480 and GAPDH (6C5): sc-32233 were from Santa Cruz Biotechnology (Dallas, TX, USA).

***Bacterial strain and cell culture***

*Escherichia coli* Nissle 1917 (serotype O6:K5:H1) was extracted from the probiotic preparation Mutaflor® as its active component. HT-29 cells were cultured at a seeding density of 2.0×10^4^ cells/cm^2 ^in 6-well plates containing 2 ml of RPMI-1640 media, which was supplemented with 10% FBS and penicillin/streptomycin (0.1 mg/ml) and kept in a humidified incubator with 5% CO_2_. Upon reaching 60–70% confluence, the cultivated cells were first washed with PBS (×3). The cells were then treated with the conditioned media (CM) and heat-inactivated bacteria (HIB). 

***Conditioned-media and heat-inactivated bacteria preparation***

For the preparation of CM, the overnight culture of bacteria (EcN) in LB broth was centrifuged at 2800 ×g for 5 min. Then, the bacterial pellets were washed with PBS (×3). The HT-29 cells cultivated in RPMI medium without antibiotics were introduced to about 0.5 McFarland concentrations of bacteria for 4–6 hr. Then, co-culture media was centrifuged at 2800 ×g for 5 min and filtered using a 0.2 μm filter. The resulting media was considered as CM and exploited for the treatment of the HT-29 cells at different time points. For production of HIB, 0.5 McFarland concentrations of an overnight culture of bacteria were heated at 70 ^°^C for 10 min, then transferred to -70 ^°^C for 5 min. These actions were repeated three times, and then the resultant bacteria were subjected to LB medium to confirm the lack of bacterial growth.

***MTT assay***

The MTT assay was utilized to determine the probable toxicity of EcN against CRC (12). Briefly, the confluent HT-29 cells in the 96-well plate were treated with a series of designated concentrations of EcN for 24 and 48 hr. Subsequently, an equal volume of DMSO (0.5%) and MTT solution (5 µg/ml PBS) were included in the control and test groups. The plate was further incubated at 37 °C. Then, the formed formazan crystals through the oxidation of MTT dye were dissolved in 200 μl of DMSO per well. Finally, the absorbance of the samples was read at 570 nm using an ELX808 UV universal microplate reader (BioTek Instruments Inc., Vermont, USA). The experiments were performed in triplicate.

***Detection of apoptotic cells by DNA fragmentation and DAPI staining assays***

DNA ladder test was used for the evaluation of DNA fragmentation and detection of apoptotic cells in the treated HT-29 cells by means of a method established previously(13). For DAPI staining, the HT-29 cells were seeded in 6-well plates containing coverslips and treated with CM or HIB for 48 hr. Afterward, to fix the cells, they were incubated with 4% paraformaldehyde for 10 min. Then, the fixed cells were washed (×3) with PBS. The samples were permeabilized by means of 0.1% Triton X-100 for 10 min. After washing (×3) with PBS, the samples were stained using 4`, 6-diamidino-2- phenylindole (DAPI) at room temperature for 5 min. The morphology of the cells was assessed using the IX81 inverted fluorescent microscope (Olympus Corp., Tokyo, Japan). The images of the cells were acquired with an Olympus DP72 digital camera.

***Flow cytometry analysis***

The annexin V-FITC apoptosis detection kit was exploited to detect any emergence of the apoptosis and/or necrosis in the HT-29 cells treated with CM or HIB. Following the manufacturer’s protocol, the samples were prepared (eBioscience, San Diego, CA, USA). Briefly, cells treated with CM or HIB for 48 hr (OD_600_: 1.0) were harvested and centrifuged at 111 ×g for 5 min. Then, the cell pellets were washed with PBS and resuspended in the annexin V binding buffer. The cells were stained using annexin V-FITC for 15 min, and then, incubated at room temperature in the dark. To resuspend the cells, the PI binding buffer was used and the cells were prepared for the analysis of apoptosis. The obtained data were analyzed using Cell Quest software (Becton Dickinson, San Jose, USA). 

***RNA extraction and RT-PCR***

Total RNA was extracted from the cells by means of Trizol reagent following the manufacturer’s protocol. The RNA concentration, quantity, and quality were confirmed by a spectrophotometer (NanoDrop Technologies, Wilmington, DE, USA) using the relative absorbance ratio at 260/280. For the cDNA synthesis, 1 µg of isolated RNAs and 0.5 µM (20 pmol) primer hexamer were made to reach 12.5 µl with diethylpyrocarbonate (DEPC)-treated water, and then, incubated at 65 ^°^C for 5 min and kept on ice for 1 min. Afterward, 2 µl (1 mM final concentration) of the dNTPs mix, 2 μl 10X reaction buffer, 1 U/µl reverse transcriptase (mM LV), and 0.5 U/µl RNase inhibitor were added, and then, 20 µl total volume of reaction was reached with DEPC-treated water. The reverse transcription condition was as follows: 25 ^°^C for 10 min and 42^ °^C for 60 min and 75 ^°^C for 5 min (14). 

***Quantification of gene expression by qPCR analysis***

The quantitative expression of mRNA of the target genes was accomplished by means of SYBR^®^ Green Master Mix on Bio-Rad IQ5 Cycler (Bio-Rad, Hercules, CA, USA). Table 1 represents the sequences of primers used in this investigation. The qPCR was fulfilled as 20 μl reaction, containing 1 μl cDNA, 10 μl of Power SYBR Green PCR Master Mix (2X), 0.2 µM from each forward and reverse primers and 8.9 μl DEPC-treated water. The PCR reaction was done based on a cycle, including 95 °C for 30 sec, followed by 40 cycles of denaturation at 94 °C for 15 sec, annealing at 60 °C for 30 sec and extension at 72 °C for 30 sec. The endogenous control GAPDH (house-keeping gene) was used for the normalization of the differences in the efficiency of RT. All experiments were repeated in triplicate (15). 

***Western blotting analysis***

The total protein was extracted from the HT29 cells using the PEB solution (i.e., 50 mM Na_2_HPO_4_, pH 7.0, 10 mM Na_2_EDTA, 2 mM β-mercaptoethanol, and 2 mM phenylmethylsulfonyl fluoride). Then, 30 µg of the extracted proteins was exposed to 12% SDS-PAGE gel. Afterward, the proteins were transferred electrophoretically to a nitrocellulose membrane and run on a semi-dry blotting system (Bio-Rad, Hercules, CA, USA). The membranes were then blocked with nonfat skim milk 5% for 12 hr. Subsequently, the membrane was incubated with the designated primary antibodies (using a 1:1000 dilution) and detected by the secondary rabbit anti-human IgG-alkaline phosphatase conjugate (1:1000) (16).

***Statistical analysis***

In this study, data were presented as the mean values with standard deviation (S.D.), which were obtained from at least three independent experiments. Student’s t-test and one-way ANOVA were utilized for the statistical analysis. A *P-*value of less than 0.05 was considered statistically significant. 

## Results

***Evaluation of treated cell morphology by CM***

**Figure 1:**
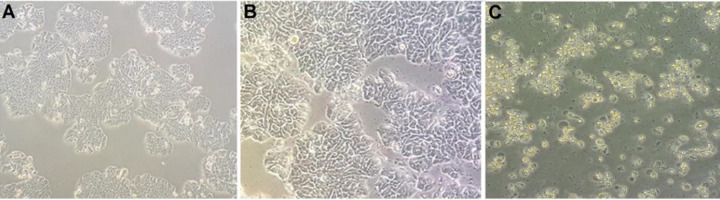
Morphological changes of HT-29 cells. The cells were treated with CM and observed under light microscopy (10X). (A) Untreated HT-29 cells (control), (B) HT-29 cells treated with CM (OD600: 1.0) after 24 hr, and (C) HT-29 cells treated with CM after 48 hr. The cells showed typical features of apoptosis including nuclear fragmentation (NF), membrane blebbing (MB), and cellular shrinkage (CS) in Figure 1C. CM: Conditioned-media

In the first step, the HT-29 cells were treated by different amounts (OD_600_: 0.5, 1.0) of CM for 24 and 48 hr. As shown in Figure 1, obvious change was observed in the cell morphology by using light microscopy imaging in (OD_600_: 1.0) after 48 hr, but no clear alteration was seen with CM (OD_600_: 0.5) after 24 hr incubation (Figure S1, supplementary data). 

***MTT assay***

**Figure 2.**
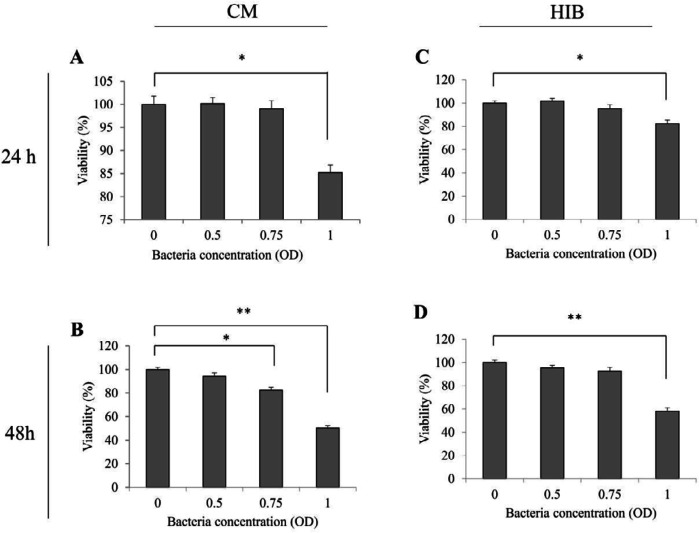
Cell viability evaluated by MTT assay in HT-29 cells treated with CM or HIB. (A) Treated cells with CM after 24 hr. (B) Treated cells with CM after 48 hr. (C) Treated cells with HIB after 24 hr. (D) Treated cells with HIB after 48 hr. Each cell viability assay was performed in triplicate. Data are presented as mean SD * and **. *P*<0.05 (*) and *P*<0.01 (**). CM: Conditioned-media; HIB: Heat-inactivated bacteria; CT: Control; OD: Optimal density

MTT assay showed the potential cytotoxic effect of EcN on HT-29 cells. For this purpose, the HT-29 cells were treated by various amounts (OD_600_: 0.5, 0.75 and 1.0) of CM and HIB for 24 and 48 hr. As presented in Figure 2, the results indicated that both treatments (CM and HIB) reduced viable cell number significantly (*P*<0.05) after 24 hr in (OD_600_: 1.0) and too significantly (*P*<0.01) after 48 hr in (OD_600_: 1.0). Thus, it can be deduced that EcN might affect the HT-29 cells. Such effect appeared to occur through a dose- and time-dependent mechanism. 

***DNA fragmentation assay***

**Figure 3:**
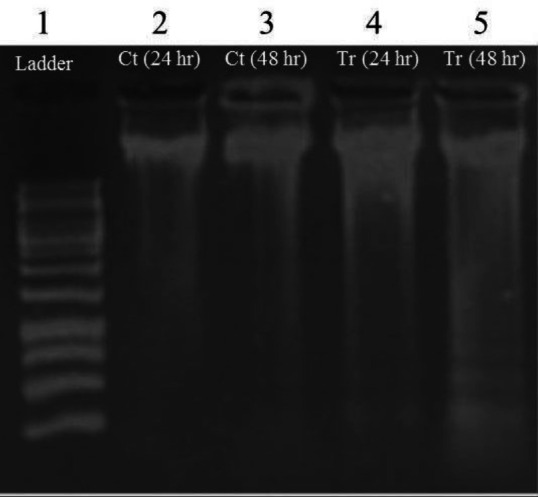
DNA fragmentation assay for detection of apoptosis. Gel electrophoresis of DNA isolated from HT-29 cells in the control and CM-treated cells. Lane 1: ladder (100bp), Lanes 2 and 3: the untreated control cells after 24 hr and 48 hr, respectively. Lanes 4 and 5: the HT- 29 cells treated with CM after 24 hr and 48 hr, respectively. Tr: Treated cells; Ct: Control; CM; Conditioned-media

Following the MTT results, the DNA fragmentation analysis was performed in the HT-29 cells treated with the CM after 24 and 48 hr. A 100 bp DNA ladder was used to verify the DNA pattern in the gel electrophoresis. In agreement with the MTT results, an apparent DNA breakage was observed after 48 hr, but not after 24 hr treatment with CM (Figure 3).

***DAPI staining***

**Figure 4:**
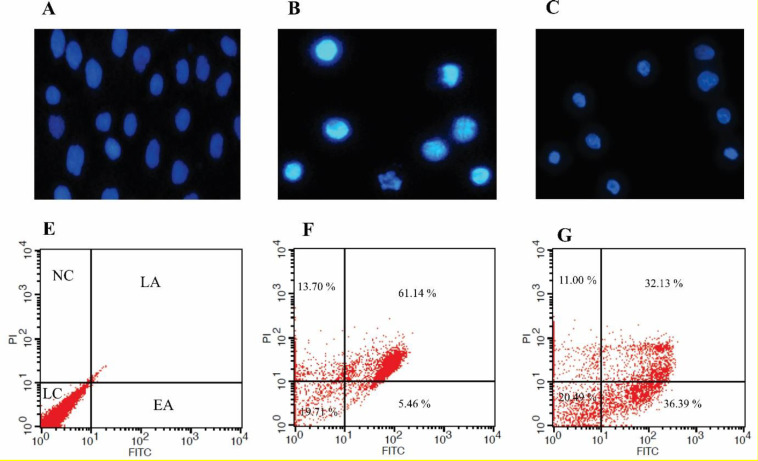
DAPI staining and flow cytometry analysis of HT-29 cells treated with CM or HIB. DAPI staining was performed to analyze changes in the nucleus. (A) Untreated control cells. (B) Treated cells with CM after 48 hr. (C) Treated cells with HIB after 48 hr. FITC-labeled annexin V/PI flow - cytometry was done to analyze apoptosis. (D) Untreated control cells. (E) Treated with CM after 48 hr. (F) Treated cells with HIB after 48 hr. CM: Conditioned-media; HIB: Heat-inactivated bacteria; NC: Necrotic cells; EA: Early apoptosis; LA: Late apoptosis; LC: Living cells

DAPI staining was carried out for the analysis of morphological changes in the nucleus. The results, in comparison with the untreated control cells (Figure 4A), displayed a substantial fragmentation and changes in the nucleus of the treated cells with CM (Figure 4B) and HIB (Figure 4C) after 48 hr. 

***Annexin V for the analysis of apoptosis***

For the differentiation of the necrotic and apoptotic cells from the healthy cells, annexin V (a phospholipid-binding protein that interacts with the externalized phosphatidylserine (PS) of the plasma membrane after apoptosis) based flow cytometry assay was performed. In comparison with the untreated control cells (Figure 4D), after 48 hr, apoptosis/necrosis was induced in the HT-29 cell treated with CM showing 5.46% and 61.14% early and late apoptosis, respectively, and 13.70% necrosis (Figure 4E). Likewise, we observed 36.39% and 32.13 early and late apoptosis, respectively, and 11.00 % necrosis in the HT-29 cells treated with HIB (Figure 4F) after 48 hr.

***Apoptosis signaling pathway regulation by EcN***

**Figure 5:**
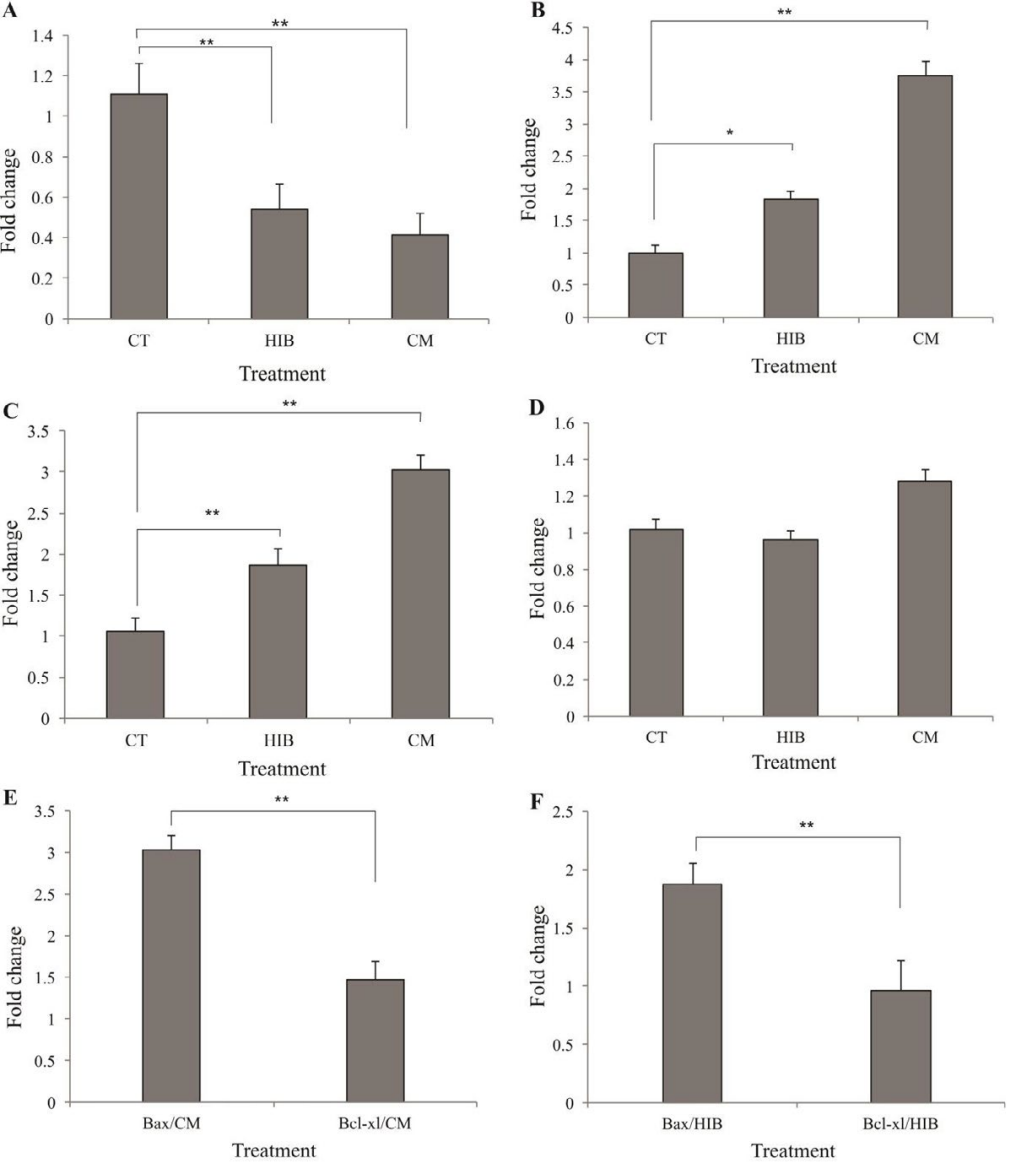
The real-time PCR analysis HT-29 cells treated with CM or HIB. The cells were treated and analyzed after 48 hr. Panels A, B, C, and D represent the mRNA expression of *AKT, PTEN, Bax*, and *Bcl-xL*, respectively. Panels E and F show the *Bax/Bcl-xL *expression ratio in the cells treated with CM or HIB, respectively. Data expressed as fold change. CT: Control; CM: Condition-media; HIB: Heat-inactivated bacteria. Data are presented as mean SD * and **. *P*<0.05 (*) and *P*<0.01 (**)

**Figure 6:**
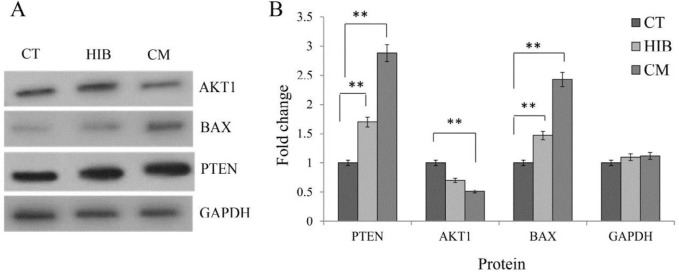
Western blot analysis of AKT, PTEN, Bax, and GAPDH proteins in HT-29 cells treated with CM or HIB. The cells were treated and probed with the indicated antibodies after 48 hr. (A) Western blot results. (B) The expression levels normalized based on GAPDH as the control. CT: Control; CM: Condition-media; HIB: Heat-inactivated. Data are presented as mean SD * and **.* P*<0.05 (*) and *P*<0.01 (**)

The Bax/Bcl-xL-triggered apoptosis via the mitochondrial pathway and AKT is involved in many cell functions, which can disrupt the balance of apoptosis and is negatively regulated by PTEN. To study the effect of EcN on the HT-29 cell signaling pathways, the impacts of both treatments (CM and HIB) were assessed after 48 hr. Based on the qPCR analysis, as shown in Figure 5, in the cells treated with either CM or HIB, the expression of the *AKT1* gene (panel A) was down-regulated, while the expressions of *PTEN* and *Bax* genes (panels B and C, respectively) were up-regulated. Similarly, the expression of *Bcl-xL* was found to be slightly enhanced in the cells treated with CM (panel D). The relative expression ratio of *Bax/Bcl-xL* was significantly higher in the treated cells with either CM or HIB (Figures 5E and F). Likewise, the Western blot analysis confirmed the down-regulation of *AKT1* and up-regulation of PTEN and Bax proteins in the cells treated with either CM or HIB as compared with GAPDH as the control (Figure 6).

## Discussion

The human gastrointestinal tract comprises a variety of complex microorganisms (microbiota) that maintain gut homeostasis (17). The significance of the microbiota interaction/association with the intestinal epithelial cells in several gastrointestinal disorders has already been reported (18, 19). Further, the therapeutic potential of probiotic strains including EcN has been indicated in several *in vitro *and *in vivo* intestinal disease models (17, 20, 21). EcN capability to exclusively colonize and replicate in the necrotic tumor tissue through systemic administration in the animal model makes this species a promising probiotic bacteria in the tumor-targeted therapy (22). Furthermore, since diets contribute significantly to the colon cancer risk factors, there exists a continuously increasing interest in the application of probiotics as a non-separable ingredient of foods and the gut microflora in colorectal cancer treatment. In this current work, we studied the anticancer effect of well-studied probiotic strain EcN on the HT-29 colorectal cancer cells by using different methods, including MTT assay, flow cytometry, DNA ladder, DAPI staining, qPCR, and Western blotting. The MTT results showed that both of the treatments (i.e., CM and HIB; OD_600_: 1.0) could effectively inhibit colon cancer cell proliferation after 48 hr but not after 24 hr (Figures 2B and D). Such a result seems to be consistent with another investigation carried out by J. Boudeau *et al*., who presented that this strain might interact with the intestinal epithelial cells possibly in a dose- and time-dependent manner (21). Based on the MTT results, in this study, other experiments were performed at (OD_600_) of 1.0 after 48 hr. Likewise, our results indicated nearly similar effects of EcN treatments (i.e., CM and HIB) on the HT-29 cells. In spite of aggressive methods for cancer treatment, the apoptotic signaling pathway can be targeted through the bacterial-targeted therapy. Prominent evidence has revealed that this process is disturbed during the progression of colon cancer, which is also related to the emergence of resistance to chemotherapy and radiotherapy (23). Hence, better understanding and targeting of this pathway seems to be a rational strategy as an alternative therapy in cancer treatment. In order to view this, we confirmed the induction of apoptosis by means of various techniques like DNA ladder DAPI staining and flow cytometry after 48 hr of EcN treatment. Furthermore, the molecular mechanism of apoptosis induction was investigated by real-time PCR analysis and Western blotting. Gene expression analysis revealed that both treatments of EcN (CM and HIB) could increase *PTEN* and decrease *AKT* activity in the treated HT-29 cells as shown in Figures 5A and B. Moreover, these findings were accompanied by Western blot analysis (Figure 6). AKT, which is a serine/threonine kinase, seems to be involved in various biological phenomena, including apoptosis, cell growth, and cell proliferation. Its activation might promote the survival of cancer cells by phosphorylating and disabling significant proteins of the apoptotic pathway, including caspase-9 and Bad, and up-regulation of anti-apoptotic Bcl-2 family proteins (14). The AKT activity is negatively controlled by the tumor suppressor protein, PTEN, which is commonly mutated in most cancer types (24). An association of phosphoinositide-3- kinase (PI3-K)/PTEN/AKT signaling pathway has been reported in the colon cancer by different research groups (25, 26). Since the expressions of *AKT* and *PTEN* were shown to be impaired in 60–70% of colorectal cancers, the inhibition of this signaling pathway has been proposed as a potential target in CRC (10). Moreover, *PTEN* up-regulation and *AKT* down-regulation were coupled with significant changes in the *Bax/Bcl-xL* ratio (Figures 5E and F). It should be pointed out that Bcl-xL is an anti-apoptotic, while Bax as a pro-apoptotic protein can associate with the intrinsic (mitochondrial) pathway of apoptosis. The *Bcl-xL *overexpression is often correlated with cancer aggressiveness and chemoresistance to therapeutics, which is negatively controlled by *Bax*. The overexpression of Bcl-2 family has been detected in about 30–94% of human CRC (23, 27). Therefore, targeting this pathway by inactivating *Bcl-xL* and activating *Bax* seems to be a promising cancer treatment. In our study, both CM and HIB treatments increased the expression of *Bax* (Figure 5C), which were further confirmed with the Western blot analysis (Figure 6). Besides, despite our expectation, the *Bcl-xL* expression was increased in the CM treatment (Figure 5D). However, the *Bax/Bcl-xL *ratio was increased in both treatments of HT-29 cells, resulting in a profound induction of apoptosis. Thus, it can be concluded that this bacterium can elicit apoptosis in the colon cancer HT-29 cells, attributable to (i) up-regulation of *PTEN*, (ii) down-regulation of *AKT*, and (iii) some possible alterations in the mitochondrial function(s). These findings are in accordance with the same study that showed EcN supernatants can effectively reduce the viability of Caco-2 cells, to 51% at 48 hr (28). Besides, it has been reported that EcN supernatant and other probiotic-derived factors can significantly decrease cyclooxygenase-2 (COX-2) gene expression (29) whose inhibition was shown to prevent the CRC initiation (30). Some shreds of evidence have shown that probiotics may play a major role in the regulation of cell proliferation and apoptosis. Iyer *et al. *reported that *Lactobacillus reuteri *might promote the apoptosis of activated immune cells by enhancing the pro-apoptotic mitogen-activated protein kinase [MAPK] signaling pathway (31). Other studies demonstrated that exopolysaccharide of  *Lactobacillus acidophilus* and *Lactobacillus. rhamnosus* exert antitumorigenic effects against HT-29 colon cancer cells possibly through the indirect induction of Bcl-2 and Bak family (32). In this line, probiotics may inhibit the progression of CRC through multiple pathways, including (i) production of specific enzymes, (ii) anti-inflammatory products, (iii) reactive oxygen species (ROS), (iv) regulation of cell cycle, and (v) apoptosis (33). Inflammation is a key factor that contributed to CRC progress and development (33). It can be estimated that EcN might regulate the expression of *AKT* by the production of anti-inflammatory products. Likewise, a prominent role of this bacterium in inflammatory disease has been indicated in a number of studies (34-36). Researchers reported that anti-inflammatory drugs (e.g., piroxicam) are able to inhibit PI3K and AKT level in colon cancer elicited by 1,2-dimethylhydrazine (37). Beside, EcN cooperates with the adaptive immune system in induction of γδ T cell apoptosis and reduction of inflammatory response (38, 39). Since metastasis remains the leading cause of death in cancer patients (40, 41), we evaluated the influence of EcN (OD_600_: 1.0) CM on the motility of HT29 cells *in vitro *(Figure S1, supplementary data). Using the scratch assay, no obvious significant difference was observed between the treated cells and the controls in terms of cell motility. However, strong inhibitory effect of *E. coli* on pulmonary metastasis has previously been reported (41). Our finding showed that EcN can induce apoptosis by modulating signaling pathways in the HT-29 CRC cells and exert anticancer effects if administrated in sufficient amount and in time. 

**Table 1:** The sequences of primers used in the qPCR procedure for analysis of various genes

Gene name	Primer Sequence (Forward)	Primer Sequence (Reverse)
*PTEN*	TCCCAGTCAGAGGCGCTATG	CACAAACTGAGGATTGCAAG
*AKT1*	CATCACACCACCTGACCAAT	CTCAAATGCACCCGAGAAAT
*Bax*	CAAACAGACCAATTCACATTT	TGTGTGCTGCTTTTGAC
*Bcl-xL*	GTTCCCTTTCCTTCCATCC	TAGCCAGTCCAGAGGTGAG
*GAPDH*	AAGCTCATTTCCTGGTATGACAACG	TCTTCCTCTTGTGCTCTTGCTGG

## Conclusion

As a complementary treatment modality, in addition to the aggressive methods in cancer therapy, the use of indigenous bacterial strains could be a promising approach to control colon cancer. Such treatment modality can balance the gut microflora, resulting in intrinsic impacts on gastrointestinal cells. Given the importance of gut microflora in human health and disease initiation, the useful properties of *E. coli *Nissle 1917 as the probiotic bacteria have been shown in GI disease. While EcN is commercially available (Mutaflor®) for the treatment of some intestinal disorders (e.g., UC, IBD, and CD), its molecular mechanisms (especially anticancer effect) are yet to be fully addressed. To tackle the therapeutic functions of EcN in CRC, in this investigation, we looked at its impacts on colon cancer HT-29 cells. Our findings revealed key information on the signaling pathways by which EcN might exert its pro-apoptotic impacts on the colon cancer cells through up-regulation of *PTEN* and *Bax* and down-regulation of *AKT1*. Moreover, similar to other probiotic strains, this bacterium might affect other cancers by modulating different signaling pathways. Based on these findings, we propose EcN as complementary medicine in cancer therapy, while further translational studies are required in animal models and human cases to revalidate its therapeutic potential in cancer inhibition and treatment. 
